# Integration of metabolite with transcript and enzyme activity profiling during diurnal cycles in *Arabidopsis *rosettes

**DOI:** 10.1186/gb-2006-7-8-r76

**Published:** 2006-08-17

**Authors:** Yves Gibon, Bjoern Usadel, Oliver E Blaesing, Beate Kamlage, Melanie Hoehne, Richard Trethewey, Mark Stitt

**Affiliations:** 1 Max Planck Institute of Molecular Plant Physiology, Science Park Golm, Am Muehlenberg, D-14476 Potsdam-Golm, Germany; 2 metanomics GmbH, Tegeler Weg, 10589, Berlin, Germany

## Abstract

**Background:**

Genome-wide transcript profiling and analyses of enzyme activities from central carbon and nitrogen metabolism show that transcript levels undergo marked and rapid changes during diurnal cycles and after transfer to darkness, whereas changes in activities are smaller and delayed. In the starchless *pgm *mutant, where sugars are depleted every night, there are accentuated diurnal changes in transcript levels. Enzyme activities in this mutant do not show larger diurnal changes; instead, they shift towards the levels found in the wild type after several days of darkness. This indicates that enzyme activities change slowly, integrating the changes in transcript levels over several diurnal cycles.

**Results:**

To generalize this conclusion, 137 metabolites were profiled using gas and liquid chromatography coupled to mass spectroscopy. The amplitudes of the diurnal changes in metabolite levels in *pgm *were (with the exception of sugars) similar or smaller than in the wild type. The average levels shifted towards those found after several days of darkness in the wild type. Examples include increased levels of amino acids due to protein degradation, decreased levels of fatty acids, increased tocopherol and decreased myo-inositol. Many metabolite-transcript correlations were found and the proportion of transcripts correlated with sugars increased dramatically in the starchless mutant.

**Conclusion:**

Rapid diurnal changes in transcript levels are integrated over time to generate quasi-stable changes across large sectors of metabolism. This implies that correlations between metabolites and transcripts are due to regulation of gene expression by metabolites, rather than metabolites being changed as a consequence of a change in gene expression.

## Abstract 2

An analysis of the temporal dynamics of metabolite and transcript levels, as well as enzyme activity, of 137 metabolites during diurnal cycles in *Arabidopsis *leaves

## Background

A full understanding of metabolic networks requires quantitative data about transcript levels, protein levels or enzyme activities, and metabolite levels. Interactions between these three functional levels will depend on the structure of the metabolic and signaling network, and on the dynamics of transcript, protein and metabolite turnover. Many inputs, including changes in metabolite levels, contribute to the regulation of gene expression. Changes in the levels of transcripts modify the levels of the encoded enzymes and the levels of metabolites or, more broadly, the metabolic phenotype. The impact of changes in transcript levels on metabolism will depend on the rates of turnover of the encoded proteins, their contribution to the control of the metabolic pathways that they are involved in, and the rates of turnover of the metabolites that are in, or are produced by, these pathways. There have been many focused studies on the impact of altered expression of single genes on protein and metabolite levels [1,2], and broader genomics studies that link changes at the levels of transcripts and proteins or enzymes [3,4], or transcripts and metabolites [5,6], but relatively few global studies of responses at all three levels [7]. Most studies have also concentrated on comparing individual conditions, rather than analyzing the temporal dynamics during a time series.

The paucity of multilevel studies is partly because of technical reasons. While global changes in expression can be routinely analyzed using custom-made or commercial arrays [8-10], it is more difficult to obtain quantitative information about the accompanying changes in protein levels and metabolites. Quantitative proteomics is still in its infancy [3,11]. The importance of analyzing changes in protein levels is underlined by the growing evidence that, at least in eukaryotes, protein levels can change independently of the levels of the transcripts that encode them [3,12]. We recently developed a robotized system to measure the activities of >20 enzymes involved in central carbon and nitrogen metabolism using optimized assays, in which the measured activity reflects changes in protein levels [4]. This platform was used to analyze changes in enzyme activities during diurnal light/dark cycles and during several days of darkness in *Arabidopsis *leaves. Most enzyme activities changed less and much more slowly than transcripts, and the attenuation and delay varied from enzyme to enzyme. Routine analysis of large numbers of metabolites is complicated by the vast number and chemical diversity of the metabolites in a given organism [13-16]. Methods have been developed for the profiling of metabolites using gas chromatography-mass spectroscopy (GC-MS) [17,18] and liquid chromatography-mass spectroscopy (LC-MS) [19] or nuclear magnetic resonance (NMR) [20,21], but to date relatively few studies have applied these technologies in combination with global analysis of levels of transcripts [5,6,22,23] or proteins [24,25].

Normalization, analysis and display of multilayered data sets also pose challenges. While considerable progress has been achieved for transcript arrays [26-28], there is no consensus on normalization strategies for metabolites and/or proteins. Typically, log fold-change normalization is used when metabolites are involved. Combined network analysis with implemented causality has been used to generate putative gene-metabolite communication networks [29] and protein-metabolite networks [30]. Deeper insights are provided when the experimental data are integrated with information about the structure of metabolic or signaling pathways, as illustrated in a recent study of glucosinolates and primary metabolism [5,6]. Although general metabolic pathway databases such as KEGG exist to support the integration of previous knowledge, it is often necessary to edit or extend them for use with a specific organism or set of organisms. Some specific plant metabolome/transcriptome pathway databases have been developed recently [16,22,31]. Software tools are also emerging that allow multiple facets of data to be displayed on a common interface [32]. However, such approaches quickly run into the limitation that only small sectors of metabolism can be usefully visualized when items are being viewed at different levels.

Plants typically grow in a diurnal light/dark cycle, providing an amenable system to analyze the temporal dynamics of changes in gene expression and metabolism. In the light, photosynthetic CO_2 _fixation drives the synthesis of sucrose in leaves and its export to the remainder of the plant to support growth and storage, whereas at night the plant becomes a net consumer of carbon [33-36]. The following experiments analyze changes in transcripts, enzyme activities and metabolites during a diurnal cycle and under two further conditions that accentuate changes in sugars; a prolonged dark treatment and the starchless *pgm *mutant. Prolongation of the night leads within a few hours to total exhaustion of starch and a collapse of sugars and related metabolites, even in wild-type (WT) plants [22]. This provides a system to investigate the responses of transcript levels, enzyme activities and metabolite levels over a longer time frame than is available in the 24 h light/dark cycle. Starch normally accumulates in leaves in the light and is remobilized and converted to sucrose at night [4,37]. The *pgm *mutant lacks plastid phosphoglucomutase activity, which is an essential enzyme for photosynthetic starch synthesis [38]. It accumulates very high levels of sugars in the day, but has very low levels of sugars in the second part of the night [36-38]. This provides a system to investigate how recurring accentuated changes in the levels of sugars impact on the diurnal responses of transcript levels, enzyme activities and other metabolites.

The responses of transcript levels and 23 enzyme activities during the diurnal cycle and an extended dark treatment in WT *Arabidopsis*, and during the diurnal cycle in starchless *pgm *mutants, were presented in [4,37]. In WT, over 30% of the genes expressed in rosettes exhibit significant diurnal changes in their transcript levels, mainly driven by changes of sugars and by the circadian clock [37]. Prolongation of the night leads to marked changes of hundreds of transcripts within 4 to 6 h [22], and thousands of transcripts after 1 to 2 days (O Blaesing, unpublished data). The accentuated diurnal changes in sugar levels in the starchless *pgm *mutant lead to exaggerated diurnal changes in the levels of >4,000 transcripts [37]. These are mainly due to the low levels of sugars at night; in the light period the global transcript levels in *pgm *resemble those in WT, whereas in the dark the global transcript profile in *pgm *resembles WT after a 4 to 8 h extension of the night [4,37]. The responses of enzyme activities were smaller and much slower than those of transcripts [4], both during diurnal cycles and the extended dark treatment in WT, and when WT is compared with *pgm*. In particular, whereas transcript levels in *pgm *resembled WT after a 6 hour extension of the night (see above), enzyme activities in *pgm *resembled WT after several days of darkness [4,22,37].

Based on these results, we propose that: changes in enzyme activities are strongly delayed compared to changes in transcript levels; and a series of transient but recurring changes in transcript levels are integrated over time as changes in enzyme activities. This conclusion is based on an analysis of 23 enzymes involved in central carbon and nitrogen metabolism. The following paper generalizes this conclusion by analyzing the responses of 137 metabolites, measured using GC-MS and LC-MS. The underlying hypothesis is that changes in the metabolite profile will integrate the responses of hundreds of enzymes across several sectors of metabolism.

## Results and discussion

### Changes in transcript levels and enzyme activities

**Figure 1:**
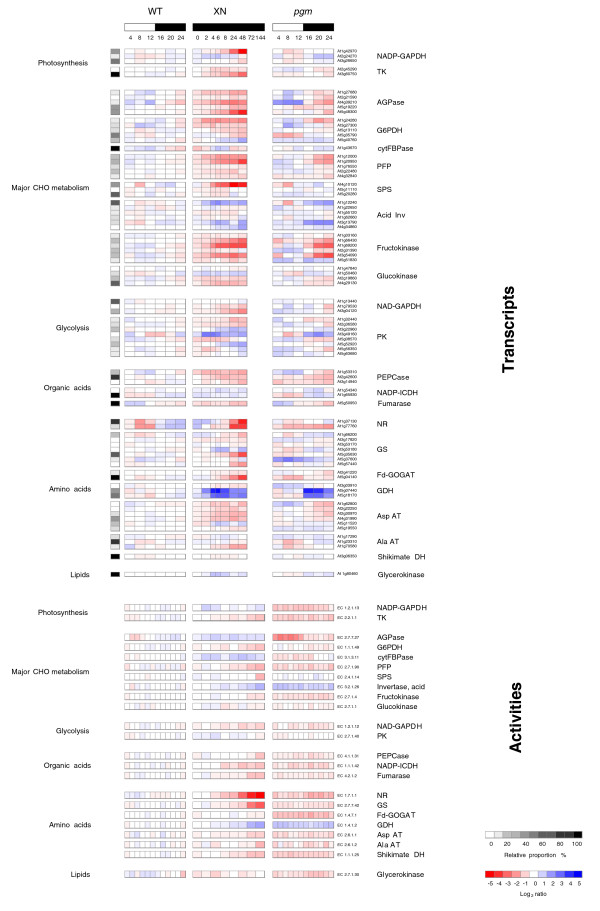
Heat map representing the changes in transcript levels and in the corresponding 23 enzyme activities in rosettes of *Arabidopsis*. Samples were taken from Col0 WT plants and Col0 *pgm *growing in a 12 h night and 12 h day cycle, throughout one day and night cycle, and in WT plants transferred to an extended night (XN). Log_2 _ratios were calculated for each value, by dividing it by the average of diurnal WT values and applying the logarithm (base 2). Log_2 _ratios give the intensity of the blue or red colors, according to the scale from the legend. Relative proportions among isoforms were calculated using the entire dataset and give the intensity of the gray color. These data are taken from [4] and [37]. CHO, carbohydrate.

A subset of the published data on changes in transcript levels and enzyme activities is summarized in Figure 1, to highlight aspects that are important for the present paper and facilitate comparison with the new data on metabolites. Figure 1 summarizes the changes in transcript levels for 82 genes, which encode the 23 enzymes analyzed in [4]. The number of genes is larger than the number of enzymes because many enzymes are encoded by small gene families. For each transcript, the average level was estimated across all the time points in WT and *pgm *diurnal cycles, and the prolonged night. These average values are shown using a monotonic color scale on the far left-hand side of the figure (the first column), and indicate which members of a given gene family are expressed at either a low or high level. A transcript level at a given time was divided by the average value, converted to a log_2 _scale and presented in a false color scale (blue = increase, red = decrease) to display the temporal changes in the transcript levels in a concise manner.

Many of the 82 genes show diurnal changes in transcript levels in WT (the second column). The amplitude and timing varies from gene to gene (Figure 1). Most show an accentuated diurnal change in *pgm *(the fourth column), including some that do not show marked diurnal changes in WT. Almost all of the genes show marked changes in their transcript levels after a prolonged night (the third column labeled XN). In most cases, the response after the prolonged night treatment represents an extension of the changes towards the end of the night in WT or *pgm*. A few genes show a change after the prolonged night that is opposite to that during the later part of the diurnal cycle in WT; for example, two genes (*NIA1*, *NIA2*) encoding nitrate reductase and one of the two genes encoding ferredoxin-glutamate synthase rose at the end of the night in WT but fell during a prolonged night. For most of these, the diurnal response in *pgm *also differs from that in WT, and the response during a prolonged night resembles that in the last part of the night in the *pgm *mutant.

The same normalization was used to depict changes in enzyme activities (Figure 1). As discussed in [4], the amplitudes of the diurnal changes of enzyme activities are unrelated to the changes of the encoding transcript levels, and the daily peak of enzyme activity is delayed compared to the peak of transcript level by an interval that varies from enzyme to enzyme. Two further aspects of the data highlight that transcript levels and enzyme activities respond with very different dynamics. First, when plants are subjected to prolonged darkness there are widespread and coordinated changes in the transcript levels for many genes within 6 h, whereas the changes in enzyme activity require several days (compare transcript levels and activities). Second, instead of showing larger diurnal changes, enzyme activities in *pgm *are typically shifted to a new value that qualitatively resembles the WT after a prolonged dark treatment. For example, transcripts for glutamate dehydrogenase and invertase show a rapid overshoot and a lower but sustained increase in WT in an extended night, and increase transiently at the end of the night in *pgm *(Figure 1). The activities rise gradually over several days in an extended night, and show a marked increase in *pgm *that is maintained across the entire diurnal cycle. An analogous response is found for many enzymes involved in respiratory metabolism, nitrogen assimilation and amino acid synthesis, including fructokinase, NAD-glyceraldehyde-3P dehydrogenase, PPi-phosphofructokinase, phosphoenolpyruvate carboxylase, NADP-isocitrate dehydrogenase, ferredoxin-glutamate synthase, alanine and aspartate aminotransferases, fumarase, shikimate dehydrogenase, and transketolase. In this case, the transcript levels fall rapidly in a prolonged night, but the activities do not decrease until several days later. Their activities during the diurnal cycle are lower in *pgm *than WT.

Our approach requires that these measurements of enzyme activity can be used as a surrogate for measurements of protein levels. In these assays, the reaction product is determined via highly sensitive enzymatic cycling systems [4], which allow the use of highly diluted extracts. All optimized assays were shown to be linear with time and independent of the extract concentration, indicating that they are not compromised by inhibitory compounds in the extracts. Substrate levels and other assay conditions were optimized to allow measurement of Vmax activity [4]. In selected cases, immunoassays were used to confirm that the changes in activity match the changes in protein level, measured by [4] (and unpublished data).

### Diurnal changes in metabolite levels in WT *Arabidopsis*

**Figure 2:**
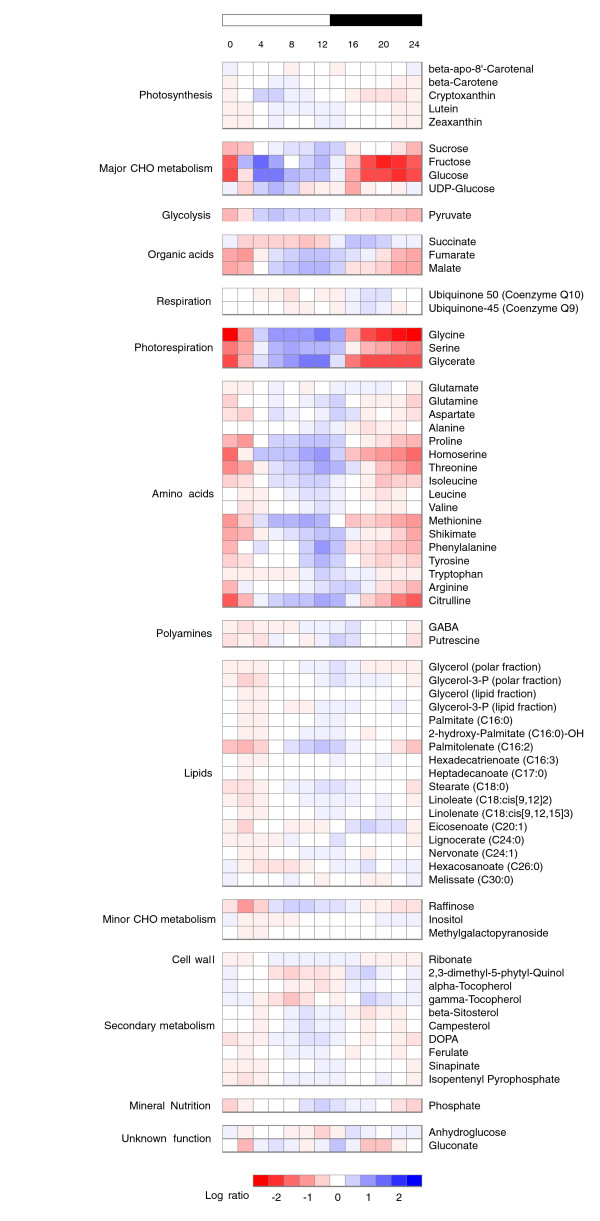
Heat map representing the changes in metabolite levels in rosettes of *Arabidopsis*. Metabolites of Col0 WT plants growing in 12 h light and 12 h night throughout one day and night cycle are shown. Log_2 _ratios were calculated for each value by dividing it by the average. Log_2 _ratios give the intensity of the blue or red colors according to the scale bar. CHO, carbohydrate.

Figure 2 summarizes the diurnal changes of 70 known metabolites during the WT diurnal cycle. The original data, including changes in 67 unidentified metabolites, are available in [Additional data file 1]. The value at each time was divided by the average level during the whole diurnal cycle; this allowed a more sensitive visualization of the changes in this data set, which were small compared to those in other conditions (see below).

A large proportion of the 137 metabolites exhibit marked diurnal changes in WT rosettes. The data were evaluated to identify metabolites that undergo authentic diurnal changes using an algorithm developed in [4], which generates a 'smoothness value' that has a value of zero if every data point lies on a smooth curve that moves through one maximum and one minimum per diurnal cycle, and increases to a maximum value of one as the data points become increasingly irregular. Using a cut-off of 0.05 as indicative of a 'good' oscillation [4], about half the metabolites showed smooth oscillations (Table 1). This includes sucrose, glucose, fructose and more unusual sugars like raffinose, all of the organic acids, glycerate, all amino acids except glutamate, which typically shows only small changes [39], glycerol-3P, many lipids (C16:2, C18:0, C18:cis[9,12]2, C20:1), many pigments and secondary metabolites, including cryptoxanthin, lutein, zeaxanthin and tocopherol, some cofactors (coenzymes Q9 and Q10), as well as many of the unidentified peaks (not shown), some of which show similar responses to known metabolites. The remaining metabolites showed more irregular responses or did not show major diurnal changes.

**Figure 3:**
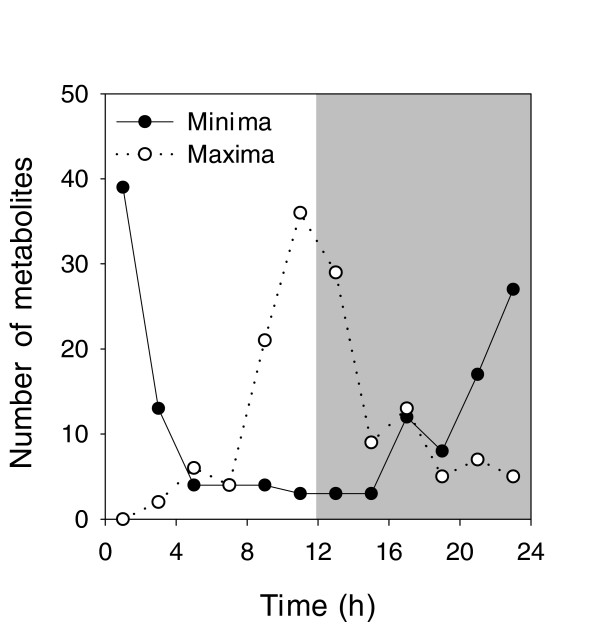
Timing of maxima and minima for metabolites across a 12 h light and 12 h night cycle, in rosettes of *Arabidopsis *Col0 WT plants. Data were smoothed prior to calculations. The shaded region indicates the dark period.

Figure 3 summarizes the frequency with which metabolites show a maximum or a minimum at different times during the diurnal cycle. A similar trend was seen, irrespective of whether this analysis was carried out with metabolites that had a smoothness value <0.05 (not shown) or all metabolites (Figure 3). Relatively few metabolites show a peak or minimum early in the light period (for example, fructose, glucose, UDP-glucose, cryptoxanthin, pyruvate) or early in the night (for example, 2,3 dimethyl-5-phytylquinol, succinate, coenzyme Q10). This would be the response expected if the metabolite level responds directly to the presence or absence of light. The vast majority peak at the end of the day, and are lowest at the end of the night (Figure 3). This is consistent with their level depending on the cumulative activity of a pathway that is active in the light. This group of metabolites included sucrose, many organic acids and amino acids, shikimate, fatty acids, glycerol and glycerol-3P.

Particularly large diurnal changes were found for sugars (sucrose, glucose, fructose), photorespiratory intermediates (glycine, serine and glycerate) and, to a lesser extent, other amino acids (Figure 2). Hexoses peaked relatively early in the photoperiod (2 to 4 h), as has also been seen in other species [33,34]. UDP-glucose peaked at 6 h and sucrose at the end of the day. Malate and fumarate rose until the end of the light period, while succinate decreased during the day and rose during the first hours of darkness (Figure 2). Accumulation of malate during the light period has been previously reported in other species, and may be related to the accumulation of malate as a counter-anion of nitrate, which decreases during the light period due to rapid assimilation of nitrate [33].

Among the fatty acids, palmitolenate (C16:2), stearate (C18:0), linolenate (C18:cis[9,12]2) and palmitate (C16:0) had a clear diurnal rhythm (Figure 2), with maxima at the end of the day and minima at the end of the night. The chloroplast contains up to 85% of the total lipids in *Arabidopsis *rosettes, mainly in the thylakoids [40], making it likely that large diurnal changes must reflect changes in this compartment. Palmitolenate (C16:2), which exhibits the strongest oscillations, is exclusively located within the chloroplast. This fatty acid is mainly present as a constituent of 1-18:2-2-16:2-monogalactosyldiacylglycerol, and is synthesized via the glycosylglyceride desaturation pathway, which takes place in the chloroplast [40].

### Changes in metabolites in a prolonged night and during diurnal changes in the starchless *pgm *mutant

**Figure 4:**
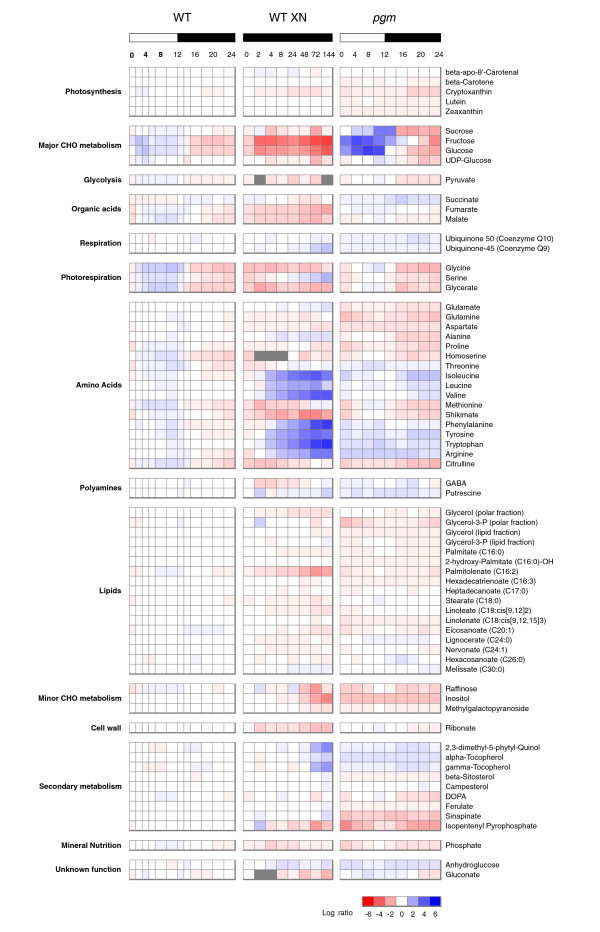
Heat map representing the changes in metabolites throughout one day and night cycle in rosettes of *Arabidopsis *Col0 WT plants, in Col0 *pgm *growing in a 12 h night and day cycle, and in WT plants transferred to an extended night (XN). Log_2 _ratios were calculated for each value by dividing it by the average of diurnal WT values and applying the logarithm (base 2). Log_2 _ratios give the intensity of the blue or red colors, according to the scale bar. CHO, carbohydrate.

Figure 4 compares the diurnal changes of metabolites in WT with the changes during the diurnal cycle in *pgm *(right-hand column) and during a prolonged night in WT (middle column). The same normalization procedure was used as for Figure 1; as a result the scale used for coloring the values in Figure 4 is different to that in Figure 2. The original data are given in [Additional file data 1].

During a prolonged night, many metabolites showed gradual but marked changes. This included a large decrease in the levels of organic acids and shikimate (an intermediate in the aromatic amino acid biosynthesis pathway), a marked decrease in C16:2 and smaller decreases in other fatty acids, including C18:0. C18:2, C18:3, and C20:1, a decrease in inositol, ribonate, gluconate and isopentenyl pyrophosphate, and a marked increase in many amino acids due to release during catabolism of proteins [22].

**Figure 5:**
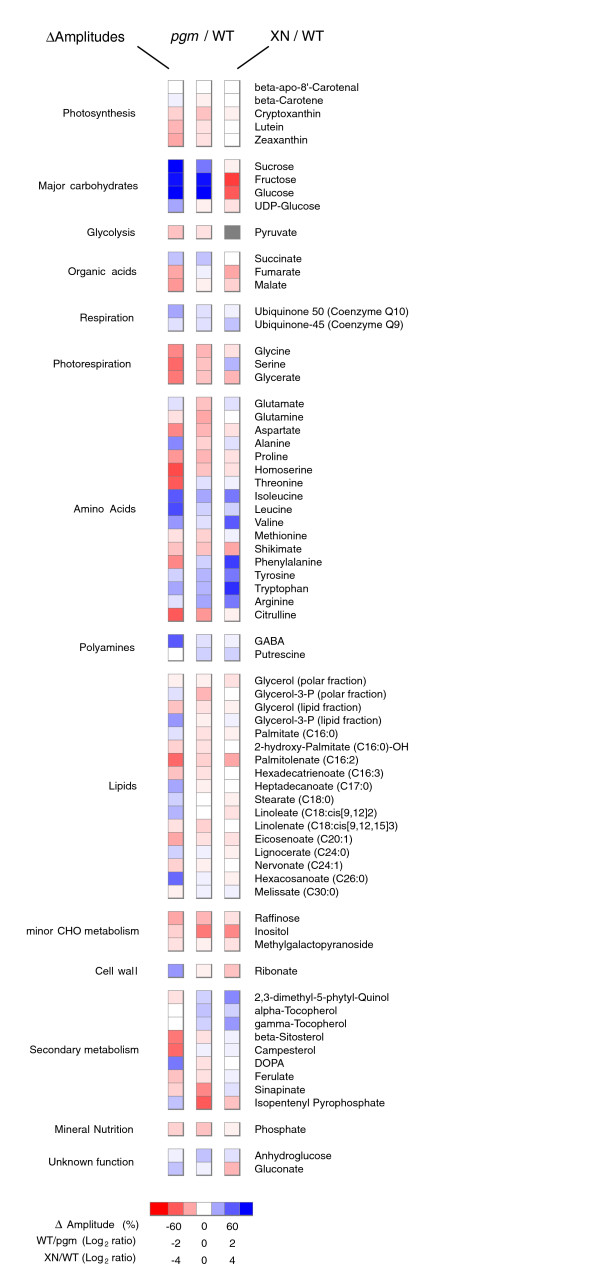
Comparative analysis of metabolite profiles obtained from *Arabidopsis *Col0 *pgm *and WT plants transferred to an extended night (XN). Heat map representing the differences in amplitudes of diurnal changes in individual metabolites calculated as maximum value - minimum value from smoothed data in WT and *pgm *(left); Log_2 _values of average (*pgm*) to average (WT) levels (middle), and Log_2 _values of average(WT) to 144 h XN (right). Scales are given in the legend.

In *pgm*, most metabolites showed similar or smaller diurnal changes than in WT (Figure 4). The left-hand column of Figure 5 uses a false color scale to highlight for each metabolite whether the amplitude of the diurnal change is larger (black-blue) or smaller (red) in *pgm *than WT. Of 137 metabolites, only 18 showed larger diurnal amplitudes in *pgm*. This included sucrose, glucose and fructose, which accumulate to high levels in the light and fall to low levels at night as a direct consequence of the lesion in starch synthesis (see Background). Seven amino acids showed a completely altered diurnal response in *pgm*, with an increase in the night instead of the day (Figure 4). This is probably due to enhanced proteolysis triggered by carbon starvation [22]. Strikingly, 32 metabolites showed smaller diurnal amplitudes in *pgm*, including some photorespiratory intermediates glycine (serine, glycerate) and several fatty acids.

**Figure 6:**
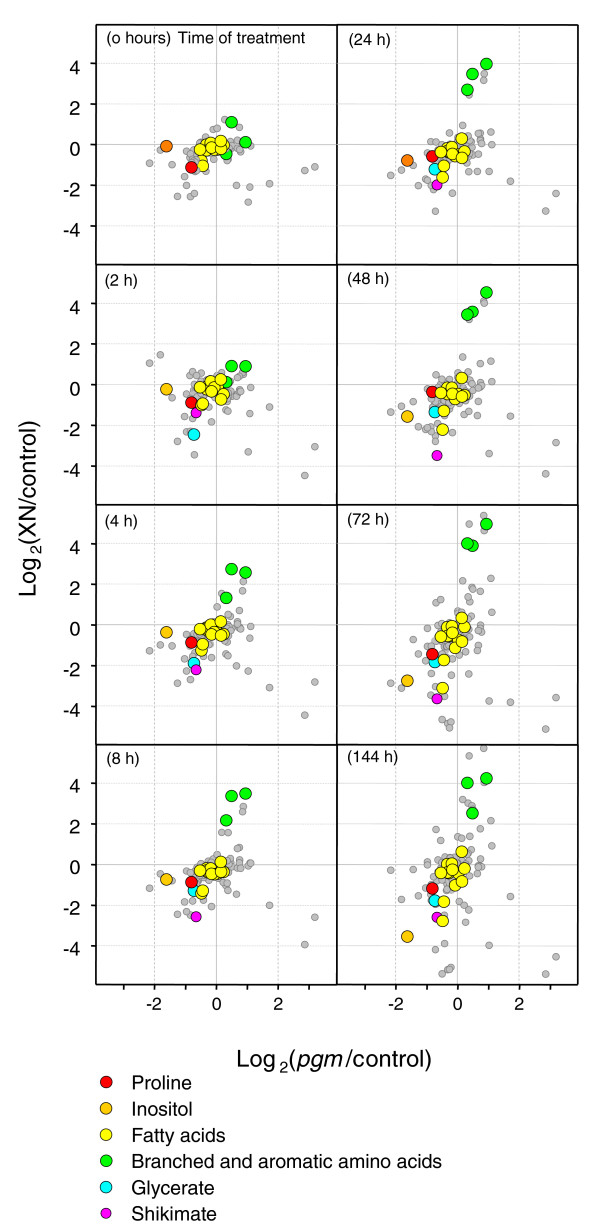
Correlation plots comparing changes in 137 metabolites in *pgm *to changes due to the extension of the night in WT. Colored plots correspond to the metabolites listed in the legend.

Figure 5 then compares metabolite levels in *pgm *with the levels in WT in an extended night. The middle column uses a false color scale to compare the average level across the diurnal cycle in *pgm *with the average level during a diurnal cycle in WT. Many metabolites show a change in their level in *pgm *(see also Figure 4). The right-hand column in Figure 5 displays the level of each metabolite in WT after 7 days of prolonged darkness, compared to the average level during a diurnal cycle in WT. Comparison of these two columns reveals that many metabolites show a qualitatively similar shift in *pgm *and an extended night. This is explored further in Figure 6 where, for each metabolite, the change between *pgm *and Col0 (x axis) is plotted against the response to an extended night in WT (y axis). With the exception of sugars, the majority of the metabolites change in the same direction in *pgm *and in an extended night in WT. This is apparent by 4 h for a subset of metabolites that increase in response to starvation, including several amino acids (Figure 4). The agreement increases with time, extending to many metabolites whose level decreases in response to starvation, like inositol, glycerate, proline, homoserine, shikimate and several fatty acids.

The *pgm *mutant is characterized by a daily alternation between elevated levels of sugars in the light, and low levels of sugars in the dark. It has already been shown that most of the genes that undergo larger diurnal changes in *pgm *are responding to the low levels of sugars in the night, rather than the higher levels of sugars in the day [37]. The finding that the metabolite profile of *pgm *leaves (with the exception of sugars and a few other metabolites) resembles that of WT plants after a prolonged dark treatment reveals that carbon starvation acts via long term mechanisms to regulate the levels of many metabolites, and generate a low-carbon metabolic phenotype. This phenotype will reflect the response of large numbers of enzymes across several sectors of central metabolism. It provides general support for the conclusions drawn from a subset of 23 enzymes in [4].

### Comparison of the amplitudes of the changes in transcript levels, enzyme activities and metabolites in diurnal cycles

**Figure 7:**
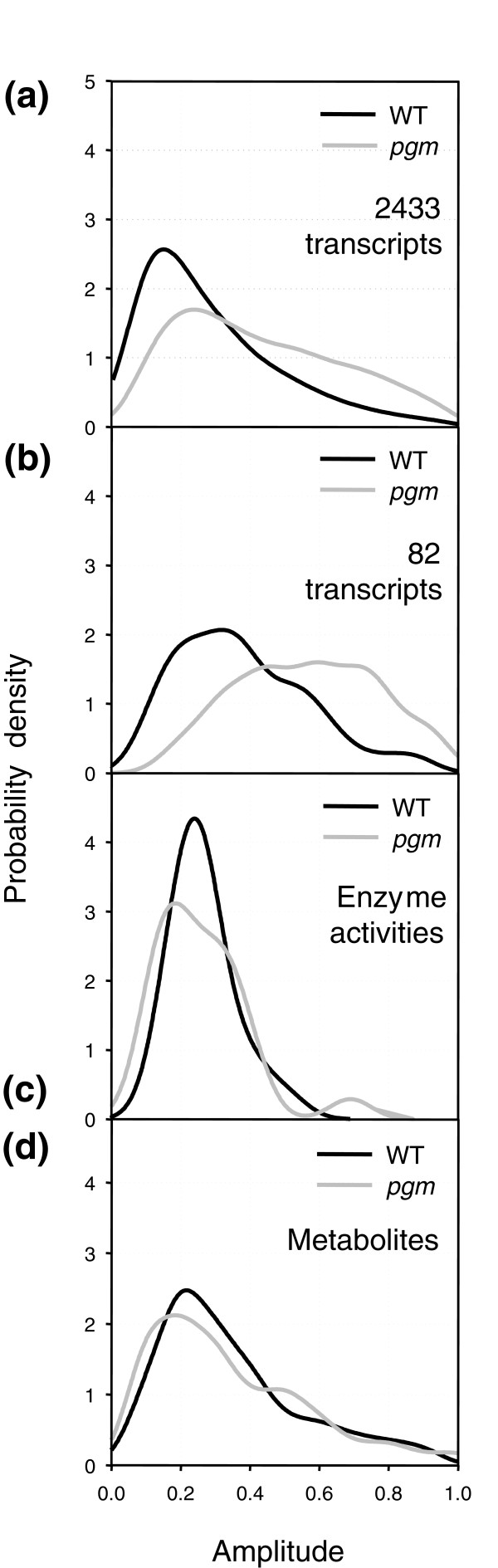
Distribution of amplitudes of diurnal changes in transcripts, metabolites and enzyme activities. Distribution of amplitudes of diurnal changes in **(a) **2,433 transcripts assigned to metabolism, **(b) **the subset of 82 transcripts encoding the enzymes measured, **(c) **23 enzyme activities, and **(d) **137 metabolites. Distributions are expressed as probability densities and were calculated with R using the function 'density', which computes kernel density estimations. The same bandwidth of 0.06 was used for all datasets.

**Figure 8:**
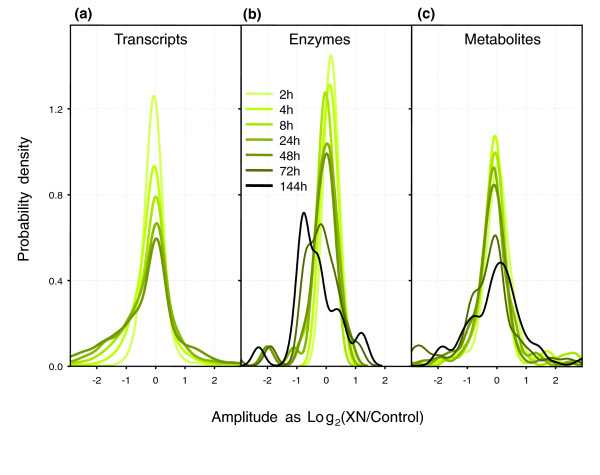
Distribution of global changes in transcript and metabolite levels and enzyme activities during the diurnal cycle and an extended night (XN). Distribution of changes in **(a) **2,433 transcripts assigned to metabolism, **(b) **23 enzyme activities and **(c) **140 metabolites after 2, 4, 8, 24, 48, 72 and 144 h of extension of the night. Transcript levels were not determined at 72 and 144 h. Distributions are expressed as probability densities and were calculated with R using the function 'density', which computes kernel density estimations. The same bandwidth of 0.2 was used for all datasets.

Comparison of the data sets for transcripts, enzyme activities and metabolites indicates that transcript levels change markedly and rapidly, whereas enzyme activities and metabolites typically change less and/or change far more slowly. These temporal dynamics are investigated more systematically in Figures 7 and 8.

Figure 7 shows the frequency distribution of the amplitudes of the diurnal changes of transcript levels for all 2,433 genes assigned to metabolism by the MapMan ontology [22,32] (Figure 7a; [Additional data file 2]), the 82 genes that encode the enzymes treated in this paper (Figure 7b), 23 enzyme activities (Figure 7c), and 137 metabolites (Figure 7d). The data for WT and *pgm *diurnal cycles are shown separately. The x axis shows the amplitude of the diurnal change (expressed as (max-min)/max), and the y-axis shows the proportion of genes that show an amplitude in that magnitude. Although current data processing of Affymetrix arrays may underestimate the extent of changes in transcript levels by a factor of two to three [41,42], this should not lead to serious error when the amplitudes are compared because this involves comparison of relative changes.

In WT, the peak values were approximately 0.15 for transcripts, and approximately 0.2 for metabolites and enzymes. The amplitudes of the changes of transcript levels were slightly larger for the 82 transcripts that encode the enzymes measured in [4] than for all 2,433 genes assigned to metabolism. The spread of amplitudes is larger for transcripts than enzymes. While most metabolites show smaller amplitudes, some show comparable diurnal changes to the most strongly responding transcripts. The *pgm *mutant has larger diurnal changes in transcript levels (approximately 0.28) but similar diurnal changes in enzyme activities and metabolites (approximately 0.2) to those in WT. There was a shift to a bi-modal distribution curve in *pgm*, with substantial numbers of transcripts, some enzyme activities and a few metabolites (mainly sugars) undergoing a diurnal change with larger amplitude. This analysis illustrates in a condensed form that large diurnal changes in transcript levels do not lead to a systematic increase of the amplitudes of the diurnal changes in enzyme activities or metabolites.

### Comparison of the temporal dynamics of the changes in transcript levels, enzyme activities and metabolites in a prolonged night

An analogous approach was taken to compare the speed and extent of the changes in metabolites, transcript levels and enzyme activities in WT during a prolonged night (Figure 8). All values were normalized on a reference value at the end of the normal night. The normalized values are shown as a series of frequency plots, which compare the amplitudes of the changes of transcript levels (Figure 8a), enzyme activities (Figure 8b) and metabolites (Figure 8c) after different times in an extended dark treatment. Figure 8a shows the changes for all 2,433 genes assigned to metabolism by the MapMan ontology. A similar result was obtained with the genes encoding the set of enzymes (not shown). After a 2 h extension of the night, a small subset of metabolites, including glucose, fructose, and glycerate, showed a marked change in their level. By 4 h, changes in transcript levels were becoming marked and by 8 h these were more widespread than the changes in metabolites. At this time, there were only minimal changes in enzyme activities. After 24 and 48 h, the changes in transcript levels became even larger and changes in enzyme activities became apparent, while the changes in metabolites became only slightly larger. Only data for enzyme activities and metabolites are available for 72 and 144 h. At these times, there were large changes in sets of enzyme activities and metabolites.

### Comparison of changes in specific transcripts, enzyme activities and metabolites

The data set was next inspected to identify examples where changes of metabolites in *pgm *or prolonged darkness can be associated with the induction or repression of specific pathway genes and/or variations in enzyme activities.

In central carbon metabolism, the accumulation of sugars in *pgm *in the light includes an increase of sucrose and a particularly large increase of glucose and fructose. The *pgm *mutant has increased levels of transcripts for most of the gene family for sucrose-P synthase, a small increase in sucrose-P synthase activity, a large increase in the levels of transcripts for two and a small increase in the levels of transcripts for another three genes encoding acid invertase, and a large increase in acid invertase activity (Figures 1 and 4). The lower levels of transcripts and activities for enzymes involved in glycolysis and organic acid synthesis in WT in prolonged darkness and in *pgm *is accompanied by lower average levels of pyruvate and, to a lesser extent, malate and fumarate. It should be noted that there are also changes in these metabolites within the diurnal cycles, and that these are not related to momentary changes in the enzyme activity (as measured in optimized conditions *in vitro*). Thus, changes in enzyme levels contribute to the mid-term shifts of metabolite levels, but are not responsible for the shorter-term changes within an individual diurnal cycle. The same holds for many of the other metabolites discussed in this section.

Qualitative agreement was also found between changes in transcript levels, enzyme activities and mid-term changes in metabolites in nitrogen metabolism. The levels of glutamine and glutamate were always lower in *pgm *than in WT *Arabidopsis *(Figure 4), as were the activities of nitrate reductase, glutamine synthetase and ferredoxin-glutamate synthase and transcript levels for the corresponding genes (Figure 1). It is known that nitrate reductase expression is regulated by sugars, acting at the level of transcription, translation and protein stability [43]. The levels of most minor amino acids, including the aromatic and branched chain amino acids, increased in a prolonged night and in *pgm*. This was associated with increased levels of transcripts for genes assigned to amino acid degradation, including *GDH *and several genes annotated as branched chain amino acid dehydrogenases [22,37], and increased glutamate dehydrogenase activity (Figure 4).

**Figure 9:**
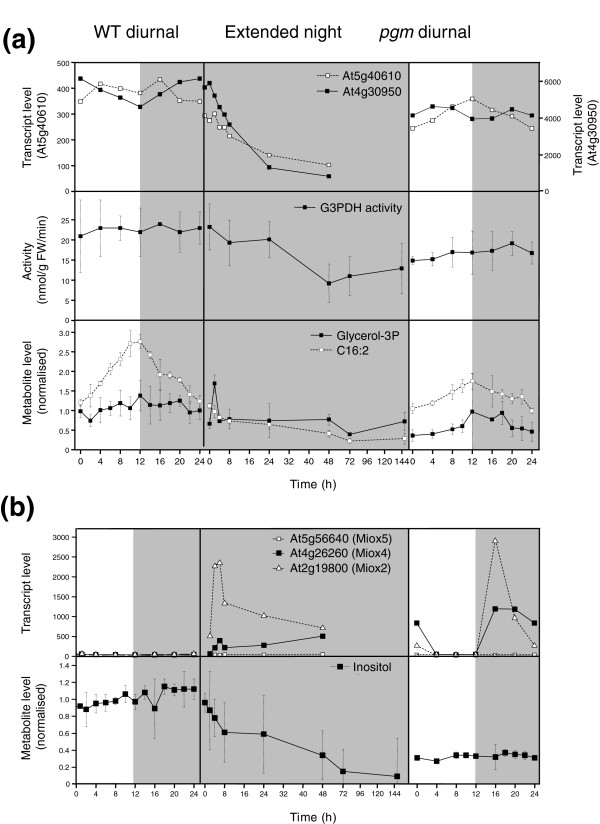
Details of specific changes in transcript levels, metabolites and enzyme activities during the diurnal cycle and an extended night. Changes in specific transcripts, enzyme activities and metabolites in rosettes of *Arabidopsis *Col0 WT plants (WT diurnal) and *pgm *(*pgm *diurnal) throughout a day and night cycle, and in WT transferred to an extended night. **(a) **Glycerolipid metabolism: levels of transcripts encoding glycerol-3P dehydrogenase (At5g40610) and fatty acid desaturase 6 (At4g30950), activity of glycerol-3P dehydrogenase, and levels of glycerol-3P and palmitolenate. **(b) **Inositol metabolism: levels of transcripts encoding inositol oxidase (At5g56640, At4g26260 and At2g19800), and inositol levels. FW, fresh weight.

Agreement between the three functional levels was also found for phospholipid biosynthesis. Several genes predicted to be involved in plastidial phospholipid synthesis [44] showed a marked diurnal cycle in the *pgm *mutant ([Additional data file 3]) and for some a strong decrease in transcript levels was observed in an extended night. For example, transcripts encoding the enzymes catalyzing the first two steps of the pathway, plastidial glycerol-3P dehydrogenase and glycerol-3P acyltransferase, showed a four-fold reduction after 48 h of prolonged night, and were also found to be lower in *pgm*. Glycerol-3P dehydrogenase activity was significantly (with a *p *value of 2E-8) decreased by 26% in *pgm *compared to WT during the diurnal cycle, and decreased gradually in a prolonged night (Figure 9a). Glycerol-3-P levels were lower in *pgm *and decreased in a prolonged night. Furthermore, these alterations were accompanied by a decrease in the levels of fatty acids in *pgm *and in WT after an extended night, especially C16:2, which is essentially contained in plastid glycerolipids (see above).

In some cases, there is agreement between the changes in the levels of transcripts and metabolites, but enzyme activities are not available to establish a clear correlation between all three levels. For example, the lower levels of inositol found in *pgm *and WT plants exposed to several days of darkness were associated with the strong induction of *MIOX2 *and *MIOX4*, which encode related inositol oxidases [45] (Figure 9b; [Additional data file 3]). The decreased levels of isopentenyl pyrophosphate observed after several days of prolonged darkness and in the *pgm *mutant were related to coherent changes in the levels of transcripts of a large proportion of genes encoding enzymes from the non-mevalonate pathway. Typically, these transcripts dropped strongly at night in *pgm*, or in WT plants transferred to a prolonged night ([Additional data file 3]). In contrast, no consistent changes were found within the mevalonate pathway. This suggests that at low carbon levels, the decrease in isopentenyl pyrophosphate synthesis mainly occurs within the chloroplast, as the non-mevalonate pathway is located in the plastids and the mevalonate pathway is in the cytosol [46].

In other cases, there are discrepancies between the functional levels. Cytosolic fructose-1,6-bisphosphatase and ADP-glucose pyrophosphorylase activity change independently of the levels of the corresponding transcripts. This discrepancy indicates that translation or degradation of these enzymes is regulated. These two enzymes and NADP-glyceraldehyde-3P dehydrogenase activity also respond differently in *pgm *and in a prolonged night, with activity being lower during the diurnal cycle in *pgm*, especially in the light, but unchanged or even increased in a prolonged night (Figure 1). One possibility is that the high sugar levels during the light period in *pgm *inhibits translation and/or promotes degradation of these proteins. Another example relates to shikimate dehydrogenase: lower levels of shikimate in *pgm *and in a prolonged dark treatment correlate with decreased activity of shikimate dehydrogenase, but there are no marked changes in *SDH *transcript levels. This indicates that post-transcriptional mechanisms contribute to the regulation of this enzyme.

There are also cases where discrepancies are already apparent, even though only two of the three functional levels have been analyzed. The increase in tocopherols in extended darkness and in *pgm *could not be related to any clear change at the level of transcripts for genes involved in tocopherol synthesis (data not shown). A similar picture emerged for ferulate, which decreased in a prolonged night and was lower in the *pgm *mutant. In these examples, measurements of enzyme activity or protein will be needed to define whether the changes in metabolites are due to translational or post-translational regulation.

### Comparison of the global relationship between metabolite levels and transcript levels

The data set was also analyzed to detect correlations between metabolite and transcript levels. The relatively slow response of enzyme activities and most metabolites to changes in transcript levels indicates that most correlations during short term responses will be due to regulation of gene expression by metabolites, rather than vice versa.

**Figure 10:**
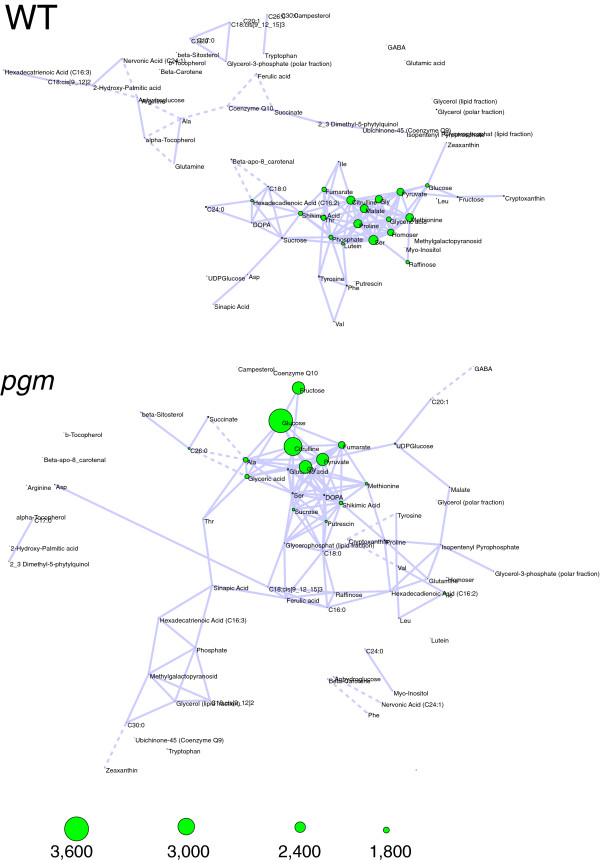
Metabolite correlation networks resulting from diurnal changes. Correlation networks of 137 metabolites determined across a day and night cycle in *Arabidopsis *Col0 WT plants and *pgm *growing in 12 h day/12 h night cycles. Metabolites with significant correlations are linked together; green discs represent the number of transcripts correlated to a given metabolite. Solid lines indicate positive correlations, dotted lines indicate negative correlations.

We first compared the changes in levels of metabolites and transcripts during the diurnal cycle. The first step in the analysis involved calculation of Pearson's correlation coefficients between metabolites during diurnal cycles in WT or *pgm *(Figure 10). These are visualized as a correlation network. Several features of the network mirrors known functional relationships. For example, glucose and fructose were connected, as were a set of intermediates from the photorespiratory pathway (glycine, serine, glycerate). The next step was to search for correlations (Pearson) between metabolites and the diurnal changes in transcript levels for all the genes on the ATH1 array. The number of genes that correlated with a metabolite (p < 0.01) is represented by the size of the green circle (see figure legend for scale). The number of transcripts that were correlated to sugars increased dramatically in *pgm *(Figure 10).

**Figure 11:**
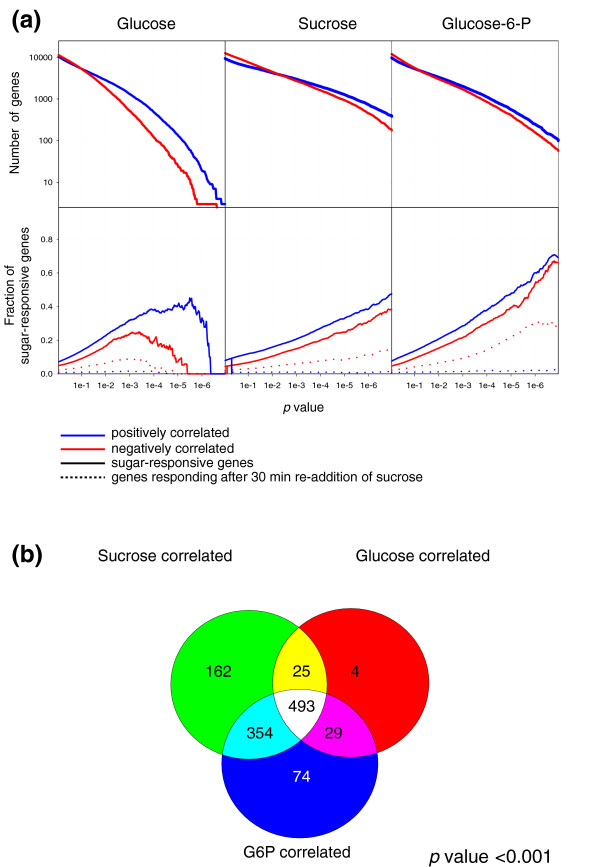
Identification of robust sugar-responsive genes whose transcripts correlate with changes in glucose, sucrose or glucose-6-phosphate during diurnal cycles in WT plants and *pgm*, and in an extended night treatment. **(a) **Correlation coefficients and corresponding *p *values (x axis, logarithmic scale) were calculated between the transcript levels for each of these genes and the levels of glucose, sucrose, or glucose-6P using a combined dataset, including data obtained from WT and *pgm *across a night and day cycle, and WT plants transferred to an extended night. The top graphs show the number of genes whose transcripts correlate with glucose (left hand), sucrose (middle) or glucose-6P (right hand) at a significance level. The bottom graphs show the proportion of the genes that correlate with a given metabolite, which are found in a set of 'sugar-responsive' genes (thick line). A set of 1,312 'sugar-responsive' genes was defined by inspection of public domain data for experiments in which sugars were added to carbon-starved seedlings for 3 h, or leaves were illuminated for 4 h in the presence and absence of CO_2_(see main text for details). The plot also shows a similar comparison against a set of genes that are induced or repressed within 30 minutes by addition of sucrose to starved seedlings (dotted line). Blue lines correspond to positive correlations, red lines to negative correlations. **(b) **Venn Diagram of sugar-regulated genes correlated to sucrose, glucose and glucose-6-phosphate with a significance level of 0.001 or better. In total, 1,141 of the 1,312 genes correlated with at least one metabolite with a *p *value < 10^-3^.

While the analysis in Figure 10 documents a qualitative difference between WT and *pgm*, these separate data sets contain too few data points to provide highly significant *p *values for individual genes. The data sets for diurnal cycles in WT and *pgm *and for WT transferred to an extended night were, therefore, combined and re-analyzed to determine if there was a relationship between the *p *values of the correlation coefficients and selected metabolites (Figure 11a). This was done using values for sucrose, fructose and glucose that had been obtained by reanalysis using enzyme-based assays. The results obtained with fructose are not shown, as glucose and fructose were highly correlated, and, therefore, both sugars have similar correlations with transcripts. In addition, we measured glucose-6-P, an intermediate in sugar metabolism. The transcript and metabolite levels were expressed on a logarithmic scale before analyzing the correlation coefficients. A large number of genes showed a high positive or negative correlation with sucrose or glucose-6-P. Relatively few genes showed a positive, and even less a good negative, correlation with glucose. A similar trend but with slightly fewer correlations was obtained when log values of transcript levels were compared with untransformed metabolite levels. Correlations calculated between untransformed transcript values and logarithmic metabolite values gave the lowest enrichments (data not shown).

To provide independent evidence that expression of these genes may be regulated by sugars or closely related metabolites, several transcript profiling data sets from published experiments in which sugar levels were changed by several different methods were inspected to complete a list of 1,312 'sugar responsive' genes. The criteria were that the genes show: a >2-fold change after addition of 15 mM glucose or 15 mM sucrose to *Arabidopsis *seedlings that had been carbon-starved for two days; and a >2-fold change between *Arabidopsis *rosettes that had been illuminated for 4 h in the presence of ambient [CO_2_] or low [CO_2_] to prevent photosynthesis [37]. The procedure is described in [37], where it is additionally shown that about 70% of these sugar-regulated genes show diurnal changes in WT, and even more in *pgm*. A list of these genes is provided in [Additional data file 4]. Figure 11a shows, for the genes whose transcript levels correlate positively or negatively with glucose, sucrose or glucose-6-P in the combined data set, what proportion is found in this list of 1,312 'sugar-responsive' genes. There was an increasingly large overlap as the *p *value was increased (Figure 11b). At *p *values <0.001, the highest overlap was found for the genes that correlated with glucose-6-P and sucrose (847 genes, that is to say >60% of sugar responsive genes).

In a reverse comparison, we asked what proportion of the 1,312 sugar-responsive genes shows highly significant *p *values (<10^-3^) with sucrose, glucose-6-phoshopate or glucose in the combined data set. To do this, the genes showing a correlation with glucose, sucrose or glucose-6-P with a *p *value <10^-3 ^(Figure 11a) were filtered, by requiring that they should be in the list of 1,312 'sugar-responsive' genes. This operation can be performed in a spreadsheet provided in [Additional data file 4]. Of the 1,312 genes in the 'sugar-responsive' set, 426 showed positive and 125 negative correlations with glucose, 626 positive and 408 negative correlations with sucrose, and 579 positive and 371 negative correlations with glucose-6-phosphate in the combined data set. More genes correlate with changes of sucrose or glucose-6-P than with glucose. Further, sucrose and glucose-6-P induce and repress similar proportions of genes during diurnal cycles, whereas glucose acts mainly to induce gene expression (Figure 11a). In total, this analysis identified a robust core of 1,141 genes whose transcripts correlate with endogenous changes in sugars, and also changes in response to addition of sugars and to a treatment that alters the endogenous sugar level.

Hexoses are sensed via at least three pathways, including one that does not require phosphorylation, one that requires hexokinase as a sensor, and one that requires phosphorylation of glucose [47], whereas there may be separate sucrose-specific sensing and signaling pathways [48]. The sensing mechanisms for sucrose are largely unknown. There is considerable overlap between genes that correlate with sucrose or glucose-6-phosphate, but little overlap between these and the genes that correlate with glucose (Figure 11b). It is tempting to speculate that glucose and sucrose act via different pathways. To further test this hypothesis, we inspected the responses of a set of 363 genes that respond >2-fold 30 minutes after adding sucrose to carbon-starved seedlings (Osuna D, Usadel B, Morcuende R, Gibon Y, Bläsing OE, Höhne M, Günter M, Kamlage B, Trethewey R, Scheible WR, and Stitt M, unpublished). Of these 363 genes, 194 and 188 were correlated at a *p *value of <0.001 to sucrose or glucose-6-P, respectively. Only 68 genes were found to be correlated to glucose at this *p *value.

Blaesing *et al*. [37] used a set of filters to identify genes whose expression might be subject to sugar-regulation during the diurnal cycle. The correlative approach in the present paper provides a refined list of candidate genes that may be regulated by a particular sugar or their derivatives ([Additional data file 4]). To assign genes to a given sugar, we performed a ranking based on the *p *values ([Additional file 4]). With stringent *p *values below 10^-6^, 8 genes were assigned to glucose, 493 to sucrose, and 211 to glucose-6-P. Strikingly, all six sugar-regulated genes encoding trehalose phosphate synthases or phosphatases were highly correlated to sucrose and/or to glucose-6P, namely At4g17770 (TPS5), At1g70290 (TPS8), At1g23870 (TPS9), At1g60140 (TPS10) and At2g18700 (TPS11) and At2g22190 (TPP H). An over-representation analysis using Fisher exact tests as in [49] was performed using MapMan [22,32] BINs as categories to identify functional groups of genes that may respond to a given sugar. This approach was carried out with genes that were positively correlated to the various sugars at different *p *value thresholds. Genes involved in protein biosynthesis were over-represented among genes for which transcript levels are positively correlated to sugars (see [Additional data file 4] for accession codes) and genes involved in protein degradation were over-represented among the negatively correlated genes (Fisher exact test; see [Additional data file 4] for accession codes). This suggests the occurrence of tight links between sugar sensing and protein turnover. Previous studies have shown that a number of genes involved in protein synthesis are repressed and genes involved in protein degradation are induced in carbon starved *Arabidopsis *[22,50].

About 5,500 genes correlate (*p* <10^-3^) with sucrose, glucose or glucose-6-P in diurnal changes and extended night treatments (Figure 10a,b), but are absent from the list of 1,312 'sugar-responsive' genes. These correlations may be due to secondary effects, or the genes might have been excluded from the list of 'sugar responsive' genes because they do not respond in one of the treatments used as filters in compiling this list. Similarly, genes that are regulated by a sugar-related input will not be necessarily correlated to endogenous sugar levels in a given set of treatments. For some genes, sugar repression only takes places below a given threshold, which is not passed in a diurnal cycle in WT plants. For example, the transcripts encoding inositol oxidases, *MIOX2 *and *MIOX4*, show little or no diurnal variations in WT (Figure 8b), but respond strongly to carbon starvation in both WT in a prolonged night and *pgm *at night.

Further experiments are needed to validate these correlations, and establish whether they reflect a causal relationship in which metabolites directly or indirectly regulate gene expression. Use of a wider range of conditions might exclude some false positives. However, stringent validation will require additional strategies, for example, the use of reverse genetics to generate small changes in the levels of specific metabolites. A two- to three-fold decrease in protein level and enzyme activity typically has little or no impact on the pathway flux, but often leads to small shifts in the levels of the substrates, products and other ligands of the enzyme, and other closely linked metabolites [51,52]. A partial inhibition of gene expression can be obtained using techniques like antisense RNA or interference RNA. The availability of large collections of knock-out mutants may allow a general strategy to be used, in which heterozygotes are used to partially inhibit enzyme activity. For many enzymes, activity is halved in a heterozygote between the WT and a null mutant [52]. A further possibility is the use of inducible gene expression to generate small and reversible changes in the levels of specific metabolites.

A particular problem in multicellular eukaryotes is that cellular or subcellular compartmentation can mask correlations between a specific pool of a metabolite and the transcript level. For example, whereas sucrose is distributed between the cytoplasm and vacuole in leaves, the vast majority of the glucose is located in the vacuole [53]. The poor correlation between transcript levels and glucose noted above shows that vacuolar glucose is not a major signal, but it remains possible that other smaller pools of glucose in other compartments, or fluxes of glucose between compartments, act as signals. In principle, techniques are available to allow comprehensive measurements of subcellular metabolite levels [53]. However, such measurements would be very time consuming, and would not provide reliable information about minor pools due to errors in correcting for cross-contamination. Powerful technologies are emerging that use imaging techniques to measure the local concentrations of specific metabolites [54,55]. A complementary strategy would be to use reverse genetics to generate targeted changes in metabolites in specific compartments. For example, overexpression of invertase in the vacuole, the cytosol and the cell wall space can be used as a strategy to alter the sucrose/reducing sugar ratio in these different metabolic compartments [51]. Notwithstanding current limitations, the occurrence of highly significant correlations in light/dark cycles and their independent validation in independent experiments in which sugars are added or endogenous pools are manipulated by changing [CO_2_] provides an initial step in dissecting these interactions.

## Conclusion

It is not yet possible to systematically establish a comprehensive gene-protein-metabolite network in plants, due to theoretical limitations in current gene annotations and technical limitations that prevent the measurement of all enzymes and metabolites (see Background). However, analysis of the dynamics of enzymes and metabolites that are technically accessible does allow a general comparison of responses and dynamics at these different levels of metabolic function, provided enough parameters are analyzed to obtain a representative picture of the response at each functional level. In the experimental systems studied in this article, levels of transcripts and some metabolic intermediates in central metabolism show rapid changes, but the majority of the 137 metabolites investigated show slow changes, which reflect the dynamics with which changes in transcript levels lead to changes in 23 enzyme activities. These results have two important implications. First, the enzyme activity profile and the metabolite profile represent an integration, over time, of faster but more transient changes in transcript levels. This may reflect the fact that plants are subject to recurrent diurnal changes and many other irregular fluctuations with a time frame of hours, days or weeks. The temporal dynamics of enzyme turnover may be hardwired to allow the metabolic phenotype to adjust to changes when they are maintained for several days, while being largely independent of short term fluctuations or recurring diurnal changes. Second, the slow response of enzyme activities, and the resulting time delay between changes in transcript levels and changes in metabolite levels, implies that correlations between transcripts and metabolites are likely to reflect a regulatory impact of metabolites on gene expression, rather than the impact of changes of gene expression on metabolism.

Even though a comprehensive analysis is not yet possible, analysis of the responses of individual parameters at all three levels already provides some functional information. Analysis of the responses of transcript and metabolite levels identified over 1,141 transcripts, whose levels are strongly correlated with endogenous changes of sugars, and half of which could be cross-validated by analyzing how they respond to changes in levels of sugars in other experimental systems. Further experiments will be required, for example using genotypes with altered levels of specific enzymes or transporters, to generate more specific changes in the levels of individual metabolites and their subcellular pools and identify the underlying metabolic signals more precisely. Analyses of the combined changes in transcript levels, enzyme activities and metabolites also identified cases where changes in expression could contribute to the changes in metabolism, and other cases where additional regulation at the level of protein synthesis or degradation is probably required.

More generally, our results show that comprehensive information about protein levels is required to dissect the causal relationship between transcription and cellular responses. The present analysis used robotized enzyme assays as a proxy for protein levels. Enzyme activity measurements are precise, have a relatively high throughput, do not require expensive or complex infrastructure and can be used in species where comprehensive genome sequence data are not available. However, it is sometimes not possible to unambiguously link the measured enzyme activity to a single gene, and this approach is only applicable to a small sector of the genome, that is, genes that encode enzymes whose activities can be easily measured. Advances in hardware and data evaluation software are currently allowing advances in quantitative proteomics [3,12]. This will ultimately allow comprehensive analysis of the dynamics of all types of proteins, including those involved in signaling and cellular structure.

## Materials and methods

### Plant growth

*Arabidopsis thaliana *var Col0, WT, and a plastidic *pgm *[38] were grown in an 8 h day growth chamber. At least 3 weeks before their use, the plants were transferred into a small growth cabinet with a 12 h day of 160 μE and 20°C throughout the day/night cycle. Harvests of 15 plant rosettes at a time point were carried out sequentially every 2 or 4 h within a day/night cycle, or after 0, 2, 4, 8, 24, 48, 72 and 144 h in total darkness. Each sample typically contained 3 rosettes, equivalent to approximately 500 mg fresh weight. The entire sample was powdered under liquid nitrogen and stored at -80°C until its use. Each experiment was repeated two times.

### RNA isolation and expression analysis with 22K Affymetrix arrays

Isolation of total RNA, cDNA synthesis, cRNA labeling and the hybridization on the GeneChip *Arabidopsis *ATH1 genome array was done as described in [22] and recommended by the manufacturer (part no. 900385, Affymetrix UK Ltd., High Wycombe, UK). The microarray suite software package (MAS 5.0, Affymetrix) was used to evaluate probe set signals of the array. The generated data files (.cel) were the input for the software package RMAExpress, which was used to normalize and estimate raw signal intensities [56]. Normalization was performed on the entire set of transcript profiles used in the present study, that is, WT and *pgm *diurnal cycles [37], and WT transferred to 0, 2, 4, 8, 24 and 48 h of extension of the night ([Additional data file 2]).

### Extraction and assay of metabolites

Batches of 15 samples, and 5 control samples obtained from 200 Col0 rosettes grown in the greenhouse, pooled and homogenized, were used. The analysis of metabolites by GC-MS was performed as described in [17]. The LC-MS [57], analyses were performed using an Agilent 1100 capillary LC system (Agilent, Technologies, Waldbronn, Germany) coupled with an Applied Biosystems/MDS SCIEX API 4000 triple quadrupole mass spectrometer (Applied Biosystems, Darmstadt, Germany). After reversed phase HPLC separation, detection and quantification was performed in the multiple reaction monitoring (MRM) mode [58]. Results are expressed as ratios between samples and the median calculated for control samples. Sucrose, glucose, fructose and glucose-6P were extracted and measured as in [36].

### Extraction and assay of glycerol-3P dehydrogenase

Aliquots of 20 mg fresh weight were extracted by vigorous mixing with 1 ml extraction buffer. The composition of the extraction buffer was 20% (v/v) glycerol, 0.25% (w/v) bovine serum albumin, 1% (v/v) Triton-X100, 50 mM Hepes/KOH pH 7.5, 10 mM MgCl_2_, 1 mM EDTA, 1 mM EGTA, 1 mM benzamidine, 1 mM aminocapronic acid, 10 μM leupeptin and 1 mM DTT.

Glycerol-3P dehydrogenase activity was determined in microplates using a stopped assay, in which 2 μl extract as well as glycerol-3P standards prepared in the extraction buffer and ranging from 0 to 100 μM were incubated for 30 minutes in 20 μl medium containing 50 mM Hepes/KOH pH 7.5, 10 mM MgCl_2_, 0.5 mM NADH, and 0 or 1 mM dihydroxyacetone-P. The reaction was stopped with 20 μl of 0.5 M NaOH. After heating for 10 minutes at 95°C to destroy dihydroxyacetone-P [59], the wells were neutralized with 20 μl of 0.5 M HCl. Glycerol-3P was then determined as in [4].

### Statistics

Standard procedures were carried out using functions of the Microsoft Excel program. Heat maps were generated in Excel using a macro. Smoothness was calculated as described in [4]. Density plots were calculated in R [60] using the 'density plot' function. Smoothing was achieved by calculating means for two consecutive points.

Correlation networks were generated by calculating all pair-wise correlations in R and considering all pair-wise correlations with a significant *p *value (0.05) calculated as described earlier [61] as connected. The resulting network was then displayed using Pajek [62] using the Kamada layout process option. When displaying correlations of transcripts and metabolites, the number of transcripts correlated with a metabolite was indicated by the size of the metabolite token.

Genes showing a significant and strong correlation to glucose, fructose and sucrose were extracted from the list of sugar regulated genes generated by [37] using an R script. These were then tested for a deviation of biological processes defined by MapMan BINS from all approximately 1,312 sugar regulated genes using Fisher's exact test.

All scripts and macros are available upon request.

## Additional data files

The following additional data are available with the online version of this paper. Additional data file 1 lists metabolite levels in WT and *pgm *throughout a 12 h day/12 h night cycle, and in WT plants transferred to an extended night for 2, 4, 8, 24, 48, 72 and 148 h. Data are expressed as means and standard deviation (*n *= 5) of ratios to the median calculated for a set of 5 control samples. Additional data file 2 lists Affymetrix array data for the conditions described. Data are given as means of RMA-normalized data, and numbers of replicates are indicated. Additional data file 3 lists levels of transcripts encoding enzymes involved in the glycerolipid and inositol metabolism, and the mevalonate and non-mevalonate pathways. Transcript levels were determined using the Affymetrix ATH1 array.Additional data file 4 lists correlation coefficients (Pearson) calculated between sugar responsive genes and sucrose, glucose or fructose. Sugar responsive genes were retrieved from [37]. Significant correlation coefficients are indicated in bold.

## Supplementary Material

Additional data file 1 Data are expressed as means and standard deviation (*n *= 5) of ratios to the median calculated for a set of 5 control samples Click here for file

Additional data file 2 Data are given as means of RMA-normalized data, and numbers of replicates are indicated Click here for file

Additional data file 3 Transcript levels were determined using the Affymetrix ATH1 array Click here for file

Additional data file 4 Sugar responsive genes were retrieved from [37]. Significant correlation coefficients are indicated in bold Click here for file

## Figures and Tables

**Table 1:** Metabolites with smooth diurnal oscillations in *Arabidopsis *Col0 WT plants growing in 12 h day and 12 h night cycles.

Metabolite	Smooth factor
Isoleucine	0.000
Cryptoxanthin	0.000
Citrulline	0.000
Proline	0.000
Shikimate	0.000
Palmitolenate (C16:2)	0.000
Threonine	0.000
Fumarate	0.000
Glycerate	0.000
Malate	0.000
Stearate (C18:0)	0.004
Homoserine	0.004
Pyruvate	0.005
Tyrosine	0.005
Phosphate	0.005
Glycine	0.005
Glucose	0.009
Raffinose	0.009
Lutein	0.010
Methionine	0.011
Serine	0.012
Ubiquinone-50 (Coenzyme Q10)	0.013
Succinate	0.014
Alanine	0.014
Tryptophan	0.014
DOPA	0.015
2,3-dimethyl-5-phytyl-Quinol	0.015
Sucrose	0.018
GABA	0.021
Valine	0.022
Linoleate (C18:cis[9, 12]2)	0.027
Glutamine	0.028
Arginine	0.035
Phenylalanine	0.036
Glycerol-3-P (polar fraction)	0.039
Eicosenoate (C20:1)	0.040
Fructose	0.041
Ferulate	0.043
Inositol	0.045
beta/gamma-Tocopherol	0.047
Leucine	0.047
alpha-Tocopherol	0.049
Zeaxanthin	0.049

Smoothness values were calculated on data previously smoothed using the moving average method. Only known metabolites with a smoothness value below 0.05 are listed.
